# Nonsense-mediated decay machinery in *Plasmodium falciparum* is inefficient and non-essential

**DOI:** 10.1128/msphere.00233-23

**Published:** 2023-06-27

**Authors:** Emma McHugh, Michaela S. Bulloch, Steven Batinovic, Cameron J. Patrick, Drishti K. Sarna, Stuart A. Ralph

**Affiliations:** 1 Department of Biochemistry and Pharmacology, Bio21 Molecular Science and Biotechnology Institute, The University of Melbourne, Parkville, Victoria, Australia; 2 Department of Physiology, Anatomy and Microbiology, La Trobe University, Bundoora, Victoria, Australia; 3 School of Mathematics and Statistics, The University of Melbourne, Parkville, Victoria, Australia; University at Buffalo, Buffalo, New York, USA

**Keywords:** mRNA degradation, malaria, nonsense-mediated decay, intron retention, RNA-seq

## Abstract

Nonsense-mediated decay (NMD) is a conserved mRNA quality control process that eliminates transcripts bearing a premature termination codon. In addition to its role in removing erroneous transcripts, NMD is involved in post-transcriptional regulation of gene expression via programmed intron retention in metazoans. The apicomplexan parasite *Plasmodium falciparum* shows relatively high levels of intron retention, but it is unclear whether these variant transcripts are functional targets of NMD. In this study, we use CRISPR-Cas9 to disrupt and epitope-tag the *P. falciparum* orthologs of two core NMD components: *Pf*UPF1 (PF3D7_1005500) and *Pf*UPF2 (PF3D7_0925800). We localize both *Pf*UPF1 and *Pf*UPF2 to puncta within the parasite cytoplasm and show that these proteins interact with each other and other mRNA-binding proteins. Using RNA-seq, we find that although these core NMD orthologs are expressed and interact in *P. falciparum*, they are not required for degradation of nonsense transcripts. Furthermore, our work suggests that the majority of intron retention in *P. falciparum* has no functional role and that NMD is not required for parasite growth *ex vivo*.

**IMPORTANCE:**

In many organisms, the process of destroying nonsense transcripts is dependent on a small set of highly conserved proteins. We show that in the malaria parasite, these proteins do not impact the abundance of nonsense transcripts. Furthermore, we demonstrate efficient CRISPR-Cas9 editing of the malaria parasite using commercial Cas9 nuclease and synthetic guide RNA, streamlining genomic modifications in this genetically intractable organism.

## INTRODUCTION

Nonsense-mediated decay (NMD) is a conserved mRNA degradation pathway that detects and eliminates transcripts containing a premature termination codon (PTC). Destabilization of nonsense transcripts occurs in almost all studied eukaryotes. Yet despite the conservation of the three core NMD proteins (UPF1, UPF2, and UPF3b) in all major eukaryotic groups, the mechanism of NMD, especially outside of opisthokonts, is unclear. Studying NMD in diverse organisms is key to understanding how this ancient quality control mechanism has evolved.

**Fig 1:**
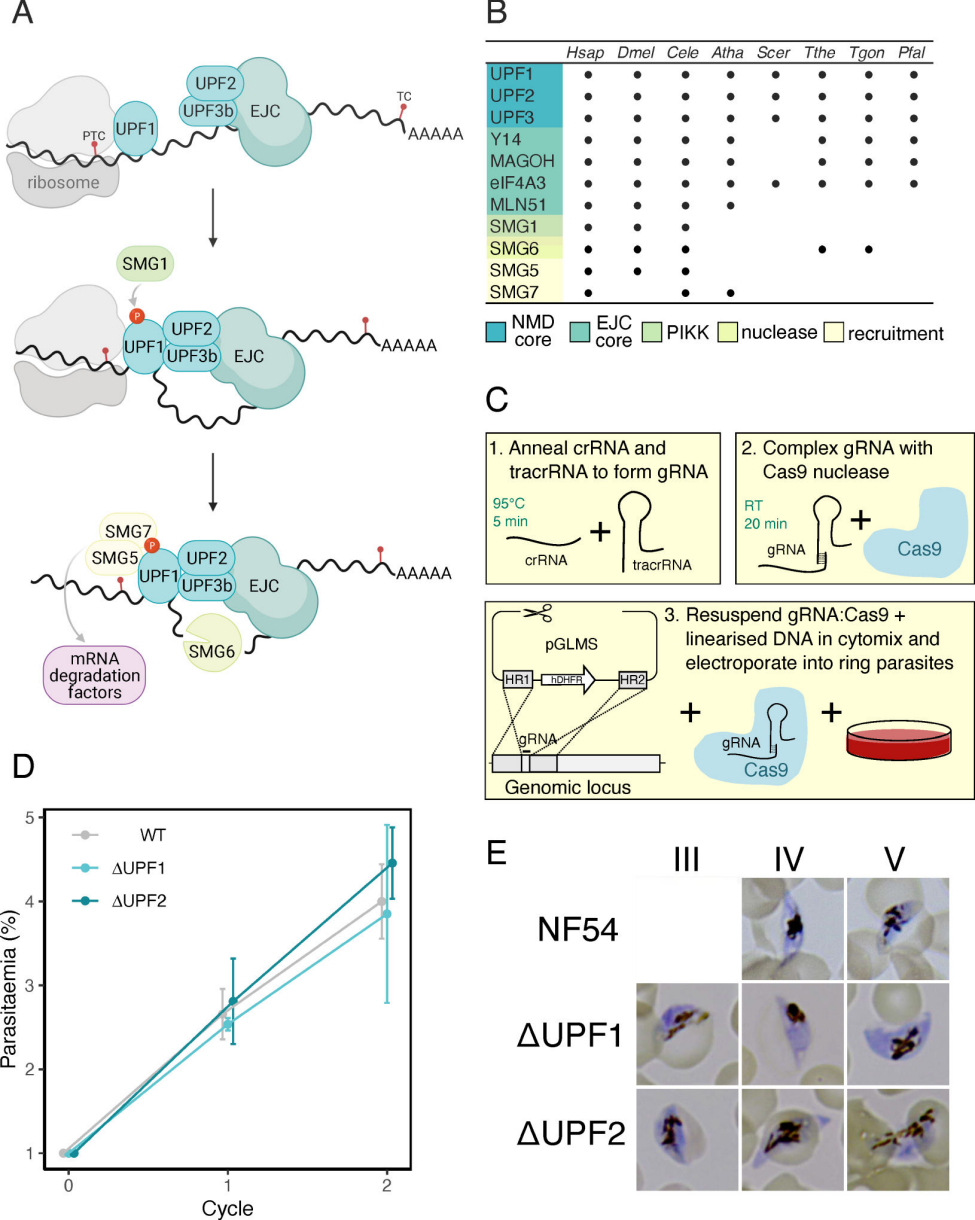
(**A**)Model of EJC-dependent NMD in humans. After ribosome stalling at a PTC, UPF2 and UPF3b associated with the downstream (>50–55nt) EJC will bind to UPF1. SMG1 phosphorylates and activates UPF1, resulting in the recruitment of SMG5 and SMG7 (involved in the further recruitment of mRNA decay factors) and the endonuclease SMG6. Drawn using biorender.com. (**B**)Presence of NMD-associated proteins in selected species (*H. sapiens*, *D. melanogaster*, *C. elegans*, *A. thaliana*, *S. cerevisiae*, *T. thermophila*, *T. gondii,* and *P. falciparum*). Proteins are grouped based on their role in NMD as determined in mammals. The NMD core is required for the initiation of NMD. EJC core proteins are deposited upstream of exon-exon boundaries after splicing and are required for EJC-dependent NMD. SMG1 is a phosphatidylinositol 3-kinase-related kinase (PIKK) which phosphorylates UPF1. SMG6 is an endonuclease. Both SMG5 and SMG7 are involved in the recruitment of UPF1 to cytoplasmic RNA degradation sites such as the exosome. This table was compiled using genes identified in reference (4). (**C**)Schematic protocol for disruption of the *Pf*UPF1 and *Pf*UPF2 genes using the Alt-R CRISPR Cas9 system (Integrated DNA Technologies). The gene-specific crRNA is annealed to the Cas9-binding tracrRNA to form gRNA (1) which is then complexed with recombinant Cas9 to form the gRNA:Cas9 RNP (2). The gRNA:Cas9 RNP is then mixed with a linearized DNA repair template encoding two homology regions (HRs) flanking a human dihydrofolate reductase (hDHFR) expression cassette. Finally, the DNA + gRNA:Cas9 mixture is electroporated into ring-stage parasites (3). (**D**)Growth analysis of WT, Δ*Pf*UPF1, and Δ*Pf*UPF2 asexual parasites. Parasite cultures were initiated at 1% parasitemia as determined by flow cytometry. Parasitemia was then measured by flow cytometry every 48h. Error bars represent SEM (*n* = 3). (**E**)Giemsa-stained WT, Δ*Pf*UPF1, and Δ*Pf*UPF2 gametocytes imaged at stages III, IV, and V of development.

During translation, ribosome stalling at a PTC can trigger NMD. There are two general models that describe how a termination codon is identified as premature and causes the initiation of NMD. These models are termed exon junction complex (EJC)-dependent NMD, which predominates in mammals, and faux 3’-UTR NMD. The former relies on EJC proteins, which are deposited 20–24 nucleotides upstream of exon-exon junctions after splicing. In mammals, EJC-dependent NMD occurs when an EJC is present > 50–55 nucleotides downstream of a PTC during translation termination (Fig. 1A) (1, 2). Faux 3’-UTR NMD is observed in animals such as *Drosophila melanogaster* and *Caenorhabditis elegans,* which have EJCs but do not require them for NMD, and also in the yeast *Saccharomyces cerevisiae*, which lacks EJC components. This model of NMD posits that after encountering a PTC, the absence of a nearby normal 3′UTR, poly(A) tail, and poly(A)-binding protein destabilizes mRNA (3). Other steps in NMD are less easily generalized by existing models due to the low conservation of proteins between eukaryotic groups. These poorly understood steps include the activation of the RNA helicase UPF1 by phosphorylation (and whether this is important for NMD in all species) and the degradation of PTC-containing transcripts by nucleases.

PTC-containing transcripts that are subject to NMD can be generated in a number of ways, including nonsense mutation, transcriptional error, or alternative splicing of pre-mRNA. Alternative splicing is widespread in eukaryotes and can refer to splicing events such as intron retention, exon skipping, or alternative splice site usage. We use the term “alternative splicing” to refer to the generation of both functional splice variants and mis-splicing/spliceosome errors. Alternative splicing frequently produces transcripts that contain a PTC, and the degradation of these transcripts by NMD has been proposed as a method for the regulation of gene expression (5, 6). This process has been best characterized by the auto-regulation of splicing factors such as serine-arginine (SR) proteins (7). Programmed intron retention leading to NMD may also contribute to global regulation of mRNA abundance in humans, yeast, and plants and has been reported to control processes such as differentiation, development, and immune responses (8 - 10).

The malaria parasite *P. falciparum* has high levels of intron retention compared to other forms of alternative splicing, which is markedly different from the proportions of alternative splice variants in humans (11). We, therefore, investigated whether intron retention coupled to NMD has a role in the regulation of gene expression in *P. falciparum*. High levels of observed intron retention could also indicate a lack of NMD. In this study, we perform CRISPR-Cas9 editing of *P. falciparum* using commercially available recombinant Cas9 and guide RNA, highlighting an efficient and cost-effective transfection strategy. We investigate the functions of the NMD core protein homologs to ascertain if *P. falciparum* has a classical NMD pathway.

## RESULTS AND DISCUSSION

### Efficient disruption of conserved NMD core proteins in *P. falciparum* using commercial CRISPR-Cas9 ribonucleoproteins

The core NMD proteins UPF1, UPF2, and UPF3 are present in diverse eukaryotes (Fig. 1B). Although the NMD core complex is highly conserved, there are some exceptions, such as in the excavate *Giardia* spp., which have retained only a UPF1 ortholog (12). Outside of the three core NMD proteins, some other proteins known to be involved in metazoan NMD have been lost in other eukaryotic lineages. For example, the EJC core proteins are required for EJC-dependent NMD in mammals; however, components of this complex have been lost in organisms that undertake EJC-independent NMD such as *S. cerevisiae* and *Tetrahymena thermophila* (13, 14). Additionally, the SMG1 kinase that phosphorylates and activates metazoan UPF1 for NMD (15) has been lost in *Arabidopsis thaliana* (but not all plants), *S. cerevisiae* (although it is present in other fungal lineages), and alveolates including *P. falciparum* (16). Despite this, global phospho-proteomics indicates that *Pf*UPF1 is phosphorylated (17) (although the kinase is unknown), and it is not clear whether this phosphorylation is important for *Pf*UPF1 activity.

To our knowledge, NMD has been studied in only two alveolates: the ciliates *T. thermophila* and *Paramecium tetraurelia*. We are interested in the parasitic phylum Apicomplexa, which encompasses parasites such as *Toxoplasma gondii* and the human malaria parasite *P. falciparum*. Previous studies have shown that *Plasmodium* spp. rely on post-transcriptional regulation for parasite development, including processes such as mRNA sequestration (18) and alternative splicing (19). Some studies have speculated that NMD coupled to intron retention—the predominant form of alternative splicing in apicomplexans—coupled to NMD could contribute to widespread regulation of mRNA abundance (11, 20), and hence, we are interested in characterizing NMD in *P. falciparum*.

We targeted two NMD genes for disruption: *Pf*UPF1 and *Pf*UPF2 (PlasmoDB IDs: PF3D7_1005500 and PF3D7_0925800). These genes were identified as the putative homologs of UPF1 and UPF2 in a bioinformatic study that cataloged RNA-binding proteins in *P. falciparum* (21). For CRISPR editing in *P. falciparum,* the Cas9 nuclease is usually encoded on a plasmid which is transfected in parallel with the homologous repair template and guide RNA expression template. Two or more of these features may be present on the same plasmid, and many variants of this strategy are currently in use with permutations of selectable markers, Cas9, guide RNA, and repair templates in different arrangements (22 - 24). However, such plasmid construction can be complex and time-consuming, and one widely adopted method for insertion of the guide RNA requires expensive reagents and special DNA purification procedures for the restriction enzyme BtgZI (25). In other cell types and organisms, an alternative to intracellular expression of the Cas9 and guide RNA is delivery of the Cas9:guide RNA complex, i.e., ribonucleoproteins (RNPs), for example, by electroporation or direct injection. RNP delivery has been used for CRISPR editing in model organisms such as *C. elegans*, *A. thaliana*, zebrafish, and mice (4, 26, 27). These RNP complexes can be delivered to human embryonic stem cells, T cells, and other cell types by electroporation (28, 29).

We made use of the Alt-R® CRISPR-Cas9 system (Integrated DNA Technologies) to disrupt the coding region of *Pf*UPF1 and *Pf*UPF2 (Fig. 1C). Custom guide RNAs were synthesized as crRNA and annealed with the Cas9-binding tracrRNA, followed by complexing with recombinant *S. pyogenes* Cas9 nuclease (Integrated DNA Technologies). The resulting RNP was electroporated into *P. falciparum* with a linear DNA repair template. Integration was confirmed by PCR, demonstrating that electroporation of *P. falciparum* with commercially available RNPs can lead to successful genome editing (Fig. S1). This method saved time on cloning of guide RNAs into unwieldy plasmids and also saved on costly plasmid assembly reagents and large-scale DNA preparation.

### NMD is not required for parasite replication or maintenance of steady-state mRNA levels

**Fig 2:**
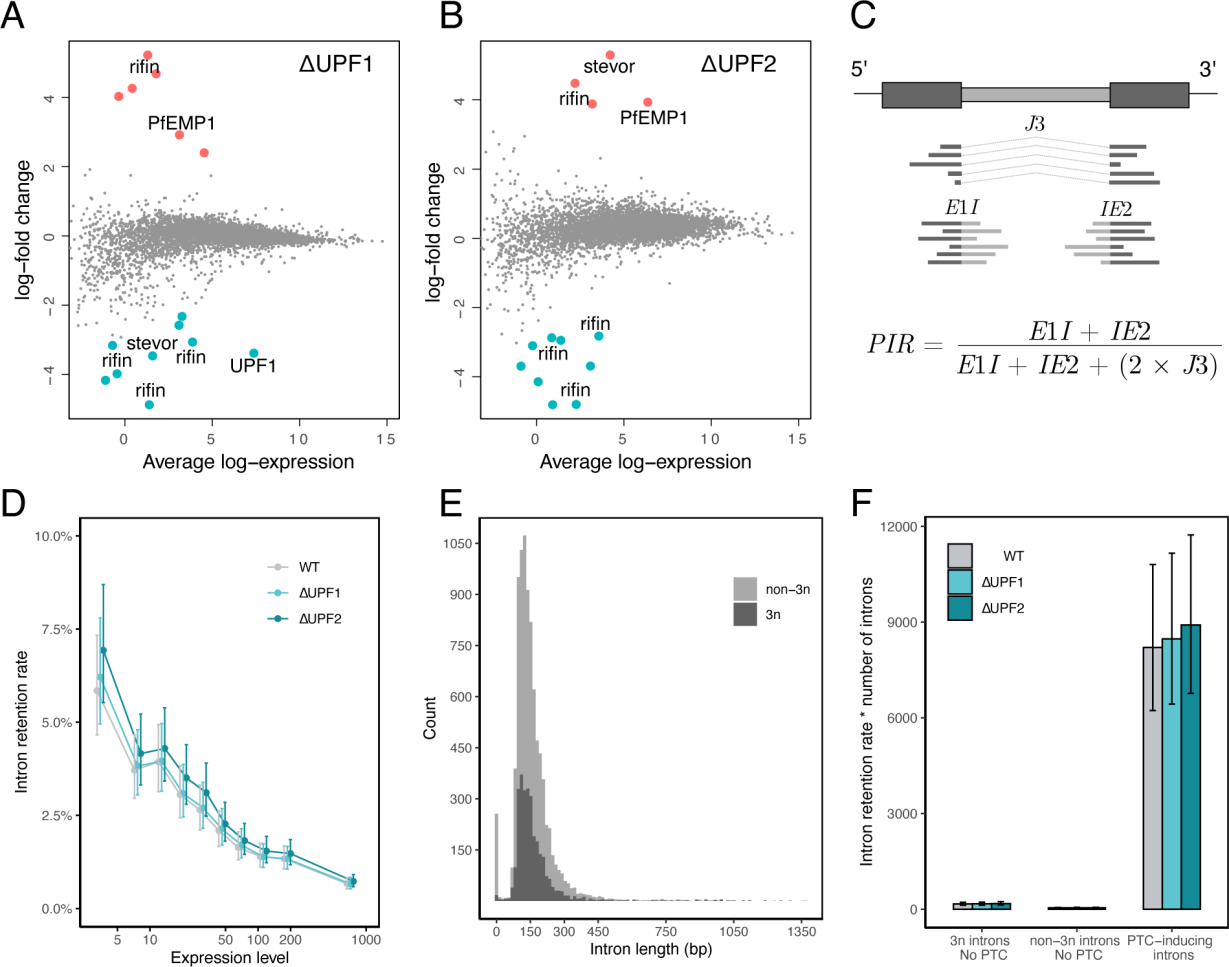
(A and B) Mean-difference plots showing differential gene expression in Δ*Pf*UPF1 and Δ*Pf*UPF2 parasites. Highlighted genes are considered significantly upregulated (red) or downregulated (blue) with an adjusted *P*-value < 0.05 (Benjamini-Hochberg) as calculated using the limma “treat” method (log-fold-change >1) with voom normalization. (C)Schematic depicting the calculation of the global proportion of intron retention (PIR). An example gene with two exons (dark gray) and one intron (light gray) is shown. *J*3 reads span the spliced junction, *E*1*I* reads span the 5′ exon-intron boundary, and *IE*2 reads span the 3′ intron-exon boundary. For a given expression level bin, reads were summed according to their designation as *E*1*I*, *J*3, or *IE*2, and the global PIR was then calculated using the formula shown. Figure adapted from the ASpli reference manual (April 27, 2020 release). (D)Introns (*n* = 7523) were grouped into 10 equal-sized bins based on expression level (WT FPKM), and the mean PIRs and confidence levels were computed using the R package emmeans within each bin for WT, Δ*Pf*UPF1, and Δ*Pf*UPF2. Error bars represent 95% CI. (E)Introns were classified into two groups: non-3n if the intron length is not a multiple of 3 (i.e., retention of the intron causes a frameshift) and 3n if the intron length is a multiple of 3. Introns were divided equally into 100 bins based on length (bp) before plotting. (F)Introns were classified into three groups based on the effect of retention: those that induce neither a frameshift nor a PTC (3n introns = 143), introns that induce a frameshift but no PTC (non-3n introns = 34), and PTC-inducing introns (6,725 introns). Intron retention was then calculated globally within each bin. For plotting, the intron retention rates and confidence intervals were multiplied by the number of introns in each of the three groups. Error bars represent 95% CI.

In keeping with a genome-wide mutagenesis study (30), we found that both *Pf*UPF1 and *Pf*UPF2 were dispensable for asexual parasite growth after disruption by CRISPR/Cas9 (Fig. 1D). Both Δ*Pf*UPF1 and Δ*Pf*UPF2 parasites were able to differentiate into sexual forms and develop into mature, morphologically normal gametocytes (Fig. 1E), indicating that these proteins are also dispensable in gametocytes development. In humans, disruption of UPF1 leads to an accumulation of transcripts containing PTCs (31). These PTC-containing transcripts can arise from transcriptional or splicing errors that would be detected by UPF1 during translation and subsequently degraded through the process of NMD. However, alternative splicing of transcripts so that they contain a PTC (e.g., intron retention or inclusion of a “poison exon”) can be important for regulation of transcript abundance. For example, some SR splicing proteins have been shown to autoregulate their expression in animals and fungi via NMD by directing alternative splicing of their cognate transcript (7, 31). We reasoned that if NMD occurs in *P. falciparum*, we would observe upregulation of transcripts modulated by alternative splicing coupled to NMD upon *Pf*UPF1 or *Pf*UPF2 disruption. To test this, we extracted RNA from WT, Δ*Pf*UPF1, and Δ*Pf*UPF2 parasites (three biological replicates) and performed Illumina mRNA sequencing. We then tested for differential gene expression using the limma-voom method. Compared to WT, there were 15 and 13 differentially expressed transcripts in Δ*Pf*UPF1 and Δ*Pf*UPF2, respectively, defined as an adjusted *P*-value < 0.05 calculated using the “treat” method with a required log-fold change >1 (Table S1). Aside from detecting downregulation of *Pf*UPF1 in the Δ*Pf*UPF1 parasites as expected (Fig. 2A), variant antigens such as *Pf*EMP1 (*P. falciparum* erythrocyte membrane protein 1), RIFINs (repetitive interspersed family), and STEVORs (subtelomeric variable open reading frame) were also differentially expressed in both Δ*Pf*UPF1 and Δ*Pf*UPF2 (Fig. 2B) parasites. These variant antigens are members of multicopy gene families that are subject to stochastic transcriptional switching (32), and so, we regard it is likely that these changes are due to switching that is independent of the disruption of NMD genes. Disruption of *Pf*UPF2 was confirmed by visualizing RNA-seq reads mapped to the wild-type genome. The limma-voom method did not automatically detect differential mRNA abundance of *Pf*UPF2 between WT and Δ*Pf*UPF2 parasites, likely due to RNA-seq reads arising from spurious transcription downstream from the disruption site that mapped to the unmodified gene, but manual inspection of these reads unambiguously revealed disruption of the wild-type transcript (Fig. S1). We also assessed intron retention in Δ*Pf*UPF1 and Δ*Pf*UPF2 parasites and found that there was no differential usage of any introns (Table S2). Together, the lack of upregulation of specific transcripts or of particular introns suggests that NMD neither does participate in targeted regulation of transcript abundance nor does produce a significant change in processing for any individual gene in asexual blood stages of *P. falciparum*.

### PTC-containing transcripts are not degraded by NMD in *P. falciparum*

One function of NMD is quality control, i.e., identifying and inducing degradation of PTC-containing mRNAs that arise from transcriptional error, nonsense mutation, and mis-splicing. This “house-keeping” role of NMD is conserved in diverse eukaryotes. It has also been suggested that the programmed generation of PTC-containing mRNAs via intron retention, and subsequent degradation through NMD, is an important method for global post-transcriptional regulation of expression (8, 10, 33). However, a study that examined intron retention from the perspective of the fitness cost of mis-splicing suggested that the vast majority of alternative splicing, including intron retention, is stochastic (34). This work, which was performed on datasets from *P. tetraurelia* and humans, showed that, in general, intron retention is inversely correlated with expression level (34). This suggests that one of the main determinants of intron retention is selection for splice site strength, as it would be disadvantageous for highly expressed genes to consume cellular resources by frequently producing aberrant transcripts. This would also imply that most intron-containing mRNAs represent splicing errors, rather than a complex program of post-transcriptional regulation as has been suggested previously (8, 34). Using this framework, we investigated the relationship between intron retention rate and expression level in WT, Δ*Pf*UPF1, and Δ*Pf*UPF2 parasites. All genes were equally divided into ten bins based on expression level (FPKM from WT), and a global intron retention rate (PIR) was calculated for all introns within each bin as shown in Fig. 2C. If NMD degrades erroneous PTC-containing transcripts, the intron retention rate should increase within a given bin when the key NMD factors are disrupted. Linear mixed-effects models indicated that there was no evidence of a difference in the intron retention rate between genotypes (Fig. 2D; *P* = 0.70) nor of an interaction between genotype and expression level (*P* > 0.99). This suggests that UPF-dependent NMD in *P. falciparum* is absent, very minimal, or non-canonical. Consistent with observations in *P. tetraurelia* and humans (34), we see an inverse correlation between intron retention and expression, arguing against widespread regulation of expression via intron retention in *P. falciparum*.

If the fitness cost of splicing errors is a main determinant of intron retention rate, we might also expect that each intron within a CDS with many introns would have a higher rate of correct (i.e., canonical) splicing than a single-intron CDS. We examined this in Δ*Pf*UPF1, Δ*Pf*UPF2, and WT parasites. As before, genes were binned by expression level, and each CDS was further sorted into three groups, depending on the number of introns it contains (1–2 introns, 3–5 introns, >5 introns). The intron retention rate was then computed globally (Fig. S2). We observed no obvious relationship between intron number and intron retention rate.

Considering that disruption of *Pf*UPF1 and *Pf*UPF2 caused no change to the overall observed intron retention rate, we next wanted to assess whether there was instead a specific effect on PTC-inducing introns. If the retention rate of PTC-inducing introns increased, but not others, this would provide evidence for the existence of a canonical NMD pathway in *P. falciparum*. Before examining the gene-disrupted parasites, we assessed intron parameters, such as length, to provide an overview of *P. falciparum* gene characteristics (Fig. 2E). Most introns induce a frameshift when retained (non-3n; 5,894 introns; median length = 139). Roughly, a third of introns have a length divisible by three and may not disrupt the reading frame when retained (3n; 2,880 introns; median length = 141). Both frameshift-inducing (non-3n) and in-frame (3n) introns can introduce a PTC when retained. We then classified each intron as either PTC-inducing or not, assuming it was the only intron retained in a given CDS. There were relatively few introns that do not induce a PTC when retained (*n* = 177) compared to those that do (*n* = 6725). The intron retention rate was then calculated globally for each category, with non-PTC-inducing introns further divided into 3n and non-3n (Fig. 2F). Linear mixed-effects models indicated that there was no evidence of a difference in the intron retention rate between genotypes (*P* = 0.47) nor of an interaction between genotype and intron category (*P* > 0.99). Overall, these results do not support the existence of a canonical NMD pathway in *P. falciparum* for the stages we analyzed.

### *Pf*UPF1 is not required for NMD

UPF1, originated in the earliest eukaryotes, is present in all major eukaryotic lineages and is highly conserved (16). Considering that UPF1 is known to be involved in NMD in some alveolates, we investigated two possibilities for the lack of effect seen in Δ*Pf*UPF1 parasites: inadvertent incorrect genetic disruption or the existence of a *Pf*UPF1 compensatory homolog.

**Fig 3:**
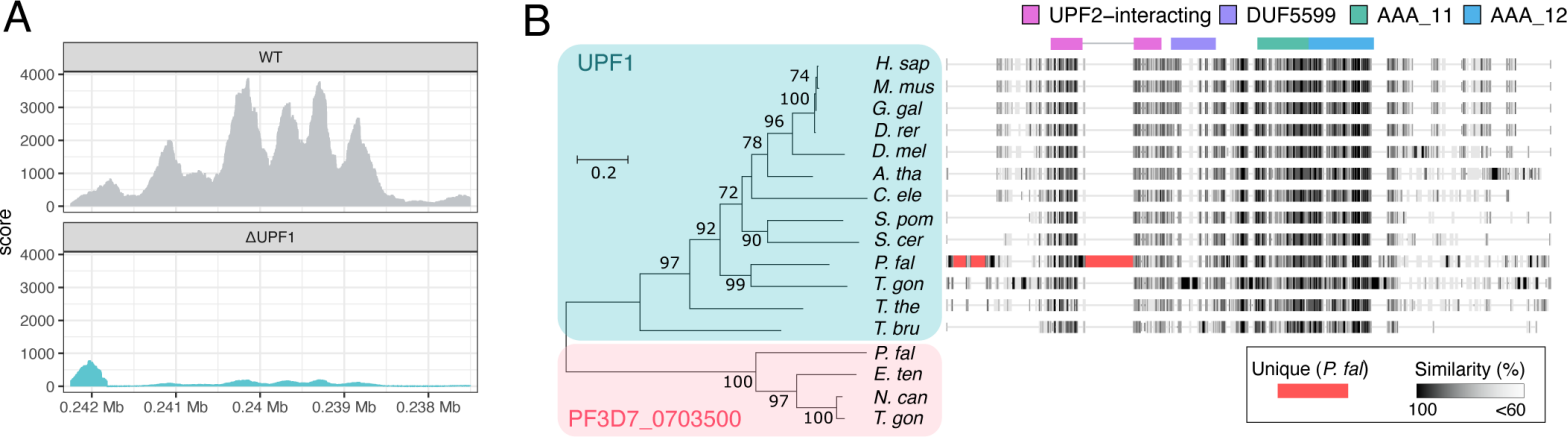
(**A**)Expression of *Pf*UPF1 in WT and Δ*Pf*UPF1 parasites. The coverage plot shows Illumina RNA-seq reads mapped to the PF3D7_1005500 (*Pf*UPF1) locus in WT and Δ*Pf*UPF1 parasites (genomic coordinates Pf3D7_10_v3: 237506–242245, negative strand). Each plot represents reads from three biological replicates. (**B**)Phylogram created with annotated UPF1 sequences (teal box) and sequences most similar to the PF3D7_0703500 protein identified by BLASTp search (pink box) from the apicomplexans *Eimeria tenella*, *Neospora caninum,* and *T. gondii*. The phylogenetic tree was constructed using the maximum likelihood method and 1,000 bootstrap replicates. Bootstrap values <50 are not displayed. Scale bar for the branch length represents the number of substitutions per site. Accession numbers and full alignments are available at https://gitlab.com/e.mchugh/nmd-paper. Conserved UPF1 domains are displayed on top of the sequence alignment. Three longer unique *P. falciparum* sequences are highlighted in red.

We first re-confirmed that *Pf*UPF1 was genetically disrupted in Δ*Pf*UPF1 parasites by examining RNA-seq reads mapping to the UPF1 locus. In Δ*Pf*UPF1 parasites, no reads overlapped the Cas9 target site, indicating complete integration of the construct in the parasite population (Fig. 3A; chromosome 10, ~0.2418 Mb). Reads mapping downstream of this site are likely due to spurious transcription initiation after the integrated drug cassette. The next possibility we considered is that the *P. falciparum* genome might contain another (as yet unannotated) UPF1. To search for such a gene, we performed an HMM search (http://hmmer.org/) using the *Homo sapiens* UPF1 sequence (UniProt ID: Q92900) as input and restricted results to the *Plasmodium* taxon. The second *P. falciparum* hit (after *Pf*UPF1) was PF3D7_0703500, which encodes a ~234 kDa protein and is currently annotated in PlasmoDB as “erythrocyte membrane-associated antigen” (35). A mutagenesis study has suggested that PF3D7_0703500 is essential and it was identified with high confidence as an mRNA-bound protein during the asexual blood stages (30, 36). Both *Pf*UPF1 and PF3D7_0703500 contain the conserved AAA ATPase domains (Pfam: AAA_11 and AAA_12) that are involved in the RNA helicase activity of UPF1 (Fig. S4). However, there is no obvious zinc-binding UPF2-interacting domain in PF3D7_0703500. The protein sequence of PF3D7_0703500 is only 24.6% identical to the *H. sapiens* UPF1, whereas there is 46.1% identity between the protein which we refer to as *Pf*UPF1 and *H. sapiens* UPF1 sequences. Additionally, a phylogram with 13 protein sequences annotated as UPF1 (Fig. 3B, teal box), plus PF3D7_0703500, and three related proteins (Fig. 3B, pink box) shows that the PF3D7_0703500-related proteins form an outgroup distinct from the UPF1s. We, therefore, consider it possible but unlikely that PF3D7_0703500 protein is having a compensatory effect for canonical UPF1 function in the Δ*Pf*UPF1 parasites. We next performed a multiple sequence alignment with UPF1 proteins from 13 species and generated a sequence similarity plot. As well as the canonical UPF1 domains (Fig. 3B, colored boxes), *Pf*UPF1 has two additional distinct regions: an N-terminal sequence (~160 residues) that is mostly distinct from the other alveolates (*T. gondii* and *T. thermophila*) and another sequence (~160 residues) within the UPF2-binding domain that is *Plasmodium*-specific (Fig. 3B, red regions in *P. falciparum* sequence alignment plot).

Given that *Pf*UPF1 had been successfully disrupted and that there is no other obvious *Plasmodium* gene that could compensate for its absence copy of the gene, we conclude that there is no UPF1 involved in NMD in *P. falciparum*. There is precedence for non-functional UPF1 in another protists—*Trypanosoma brucei* appears to have lost dependence on for degradation of PTCs while retaining a UPF1 ortholog (37). Although canonical, UPF1-dependent NMD appears absent in *P. falciparum*, other UPF components are present in *Plasmodium*, and we next examined their impact on nonsense-mediated decay.

### The core NMD orthologs co-immunoprecipitate in *P. falciparum*

**Fig 4:**
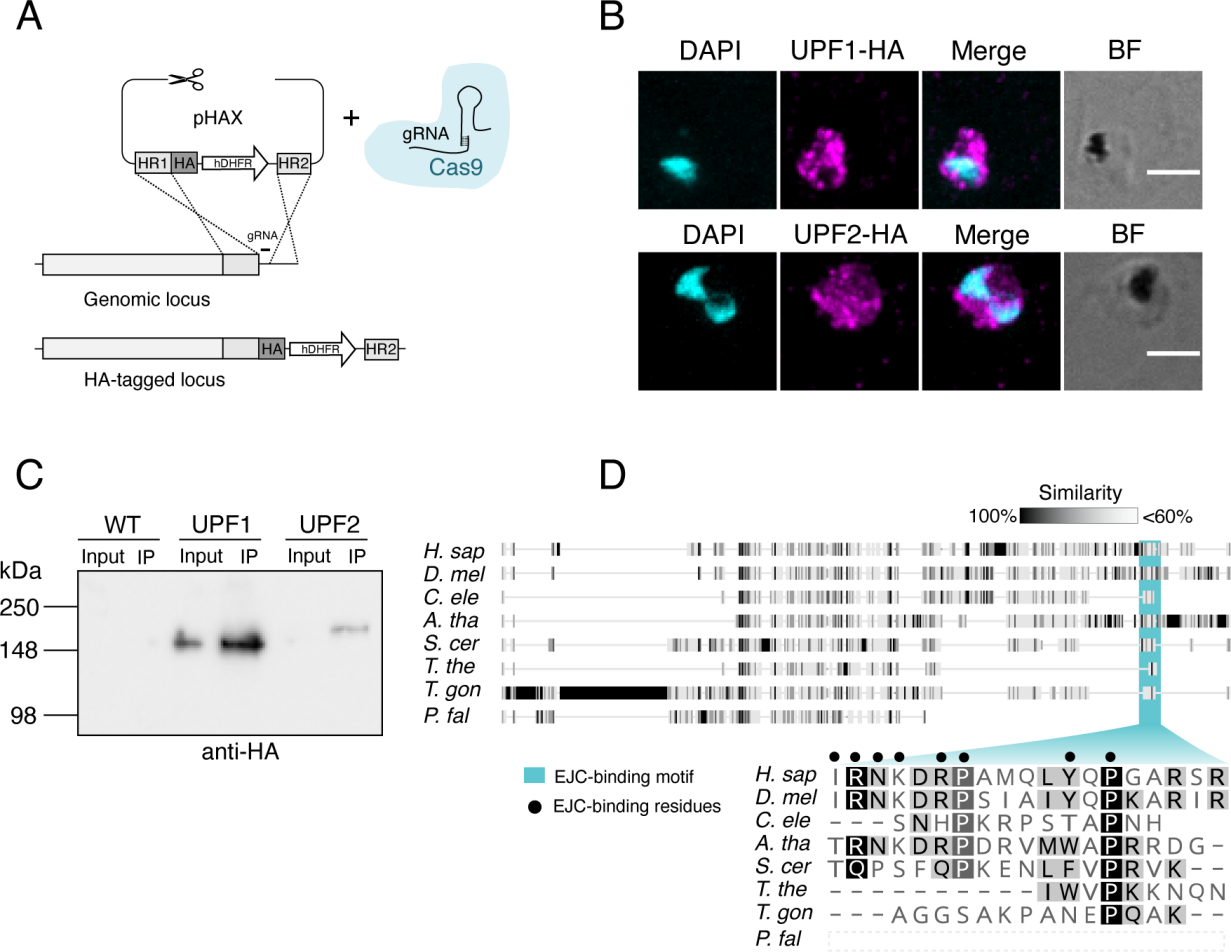
(**A**)Strategy for introducing epitope tags to *Pf*UPF1 and *Pf*UPF2. Homology regions (HRs) targeting the C-terminal of the CDS (excluding the stop codon) and a region in the 3′ UTR were cloned on either side of a 3X-HA and hDHFR drug resistance cassette in the plasmid pHAX. (**B**)Immunofluorescence assay with *Pf*UPF1-HA and *Pf*UPF2-HA parasites. Infected RBCs were fixed with paraformaldehyde/glutaraldehyde, permeabilized with Triton X-100, and probed with rat anti-HA (1:300), followed by AlexaFluor anti-rat 568 (1:600). Images are maximum projections of wide-field deconvoluted z-stacks. Nuclei were visualized with DAPI (cyan) and HA signal is presented in magenta. BF, brightfield; scale bars = 3 μM. (**C**)Immunoprecipitation and Western blot of WT, *Pf*UPF1-HA, and *Pf*UPF2-HA. For each parasite line, the input (3% of the Triton X-100-soluble fraction) was loaded beside the immunoprecipitated eluate (100% of the IP). The membrane was probed with anti-HA (1:1,000). (**D**)Alignment of UPF3b sequences. The residues in human UPF3b that are important for EJC-binding are denoted with black dots [as determined in reference (38)]. Accession numbers and full alignments are available at https://gitlab.com/e.mchugh/nmd-paper.

We used CRISPR-Cas9 as described above with a donor vector encoding a 3X-HA tag to C-terminally epitope tag *Pf*UPF1 and *Pf*UPF2 (Fig. 4A), creating the parasite lines *Pf*UPF1-HA and *Pf*UPF2-HA, respectively. Immunofluorescence microscopy of *Pf*UPF1-HA and *Pf*UPF2-HA identified the epitope tag of each protein within the cytoplasm of the parasite, with some fluorescent puncta (Fig. 4B).

We performed co-immunoprecipitation (co-IP) with anti-HA agarose beads of *Pf*UPF1-HA and *Pf*UPF2-HA. Immunoblotting showed that *Pf*UPF1-HA and *Pf*UPF2-HA migrated close to their predicted molecular weights (188 kDa and 212 kDa, respectively) (Fig. 4C). *Pf*UPF1-HA was detected in both the input and IP eluate. Although *Pf*UPF2-HA was successfully enriched and detected in the IP eluate, the concentration in the input was below detection by immunoblotting—likely due to lower expression of *Pf*UPF2. In order to discover proteins that co-IP with *Pf*UPF1-HA and *Pf*UPF2-HA, we performed LC-MS/MS on the IP eluates. Proteins were considered to be co-IPed if they were at least fivefold enriched in the *Pf*UPF1-HA (Table 1) or *Pf*UPF2-HA (Table 2) eluates compared to WT eluate, with a minimum of two significant peptides in both biological replicates. By these criteria, *Pf*UPF2-HA co-IPed with both *Pf*UPF1 and *Pf*UPF3b, indicating that the core NMD proteins interact with one another in *P. falciparum. Pf*UPF1-HA did not consistently co-IP with *Pf*UPF2 or *Pf*UPF3b although peptides from these proteins were detected in one out of two experiments (these proteins of interest are included below the double line in Table 1). As we had identified the putative RNA helicase PF3D7_0703500 by homology to UPF1, we were interested to observe that, in the *Pf*UPF2-HA samples, peptides were detected not only in both replicates but also in one of the WT controls, and hence, spectral counts are listed below the double line in Table 2 for interest. In addition to *Pf*UPF1, *Pf*UPF2-HA also co-IPed with another RNA helicase, annotated as DBP1 (PF3D7_0810600). Both *Pf*UPF1-HA and *Pf*UPF2-HA co-IP with a number of ribosomal proteins which is unsurprising as NMD is a translation-dependent process, and in other organisms, NMD factors are known to interact directly with some ribosomal components (39).

**TABLE 1:** List of proteins co-immunoprecipitated with UPF1-HA

PlasmoDB ID	Protein description	Significant MS/MS spectra			
		UPF1-HA	WT	UPF1-HA	WT
PF3D7_1005500	Regulator of nonsense transcripts 1 (UPF1)	49	NA^*a*^	11	NA
PF3D7_0322900	40S ribosomal protein S3A (RPS3A)	16	1	4	NA
PF3D7_1347500	DNA/RNA-binding protein (ALBA4)	13	1	11	1
PF3D7_1027300	Peroxiredoxin (nPrx)	10	NA	5	NA
PF3D7_0617200	BFR1 domain-containing protein (BFR1)	9	1	2	NA
PF3D7_0814000	60S ribosomal protein L13-2 (RPL13-2)	6	NA	2	NA
PF3D7_1325100	Phosphoribosylpyrophosphate synthetase (PRPS)	5	0	4	NA
PF3D7_1124900	60S ribosomal protein L35 (RPL35)	5	1	6	0
PF3D7_0905400	High-molecular-weight rhoptry protein 3 (RhopH3)	2	NA	2	NA
PF3D7_0925800	Regulator of nonsense transcripts 2 (UPF2)	4	NA	NA	NA
PF3D7_1327700	Regulator of nonsense transcripts 3B (UPF3b)	0	NA	2	NA
PF3D7_1006800	Single-strand telomeric DNA-binding protein GBP2	7	NA	0	NA

^*a*^ NA, not available.

**TABLE 2:** List of proteins co-immunoprecipitated with UPF2-HA

PlasmoDB ID	Protein description	Significant MS/MS spectra			
		UPF2-HA	WT	UPF2-HA	WT
PF3D7_0925800	Regulator of nonsense transcripts 2 (UPF2)	20	NA^*a*^	18	NA
PF3D7_0817700	Rhoptry neck protein 5 (RON5)	9	1	4	NA
PF3D7_1327700	Regulator of nonsense transcripts 3B (UPF3b)	8	NA	10	NA
PF3D7_1005500	Regulator of nonsense transcripts 1 (UPF1)	6	NA	5	NA
PF3D7_1408000	Plasmepsin II (PMII)	6	NA	2	NA
PF3D7_1006800	Single-strand telomeric DNA-binding protein GBP2	4	NA	3	NA
PF3D7_1347500	DNA/RNA-binding protein (ALBA4)	5	1	10	1
PF3D7_1323100	60S ribosomal protein L6 (60SRPL6)	2	NA	2	NA
PF3D7_1134000	Heat shock protein 70 (HSP70)	2	0	2	NA
PF3D7_0810600	ATP-dependent RNA helicase (DBP1)	2	NA	4	0
PF3D7_0719600	60S ribosomal protein L11a (RPL11a)	2	NA	2	NA
PF3D7_1002400	Transformer-2 protein homolog beta (TRA2B)	2	NA	3	NA
PF3D7_0703500	Erythrocyte membrane-associated antigen	3	3	2	NA

^*a*^ NA, not available.

No components of the EJC core complex were identified in any replicate for *Pf*UPF1-HA nor *Pf*UPF2-HA (Table S3). In humans, a conserved motif in UPF3b acts as a bridge between the NMD core and the EJC (40, 41). This EJC-binding motif is absent in *Pf*UPF3b (Fig. 4D, top panel) as this protein is C-terminally truncated compared to human UPF3b (271 vs 483 residues, respectively). The ciliate *T. thermophila*, which performs EJC-independent NMD, also lacks key residues from the human UPF3b EJC-binding motif (Fig. 4D, bottom panel) (14).

**Fig 5:**
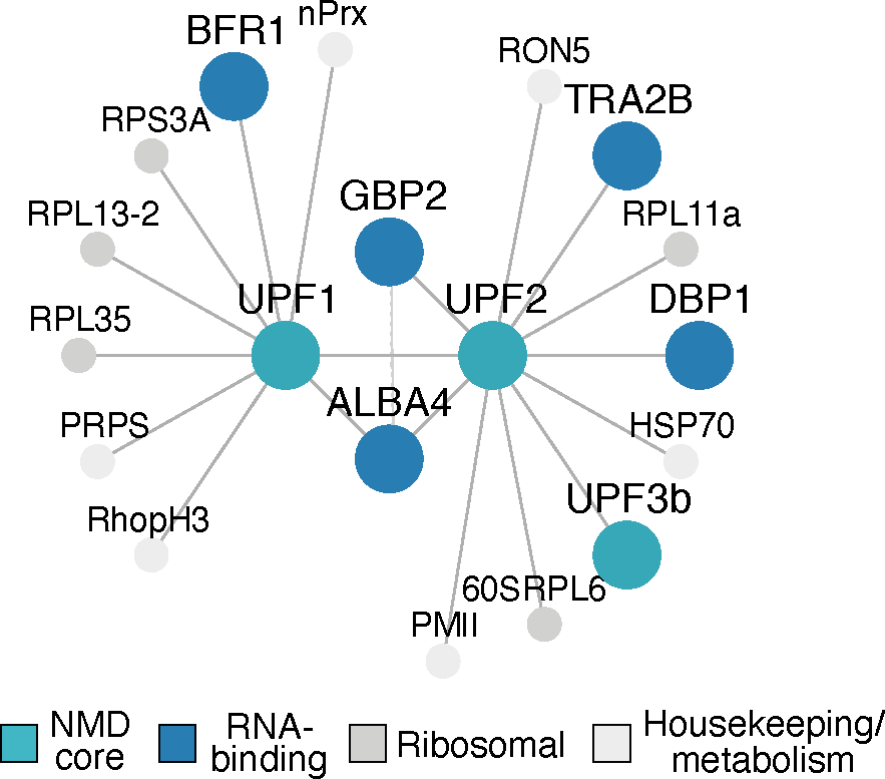
(**A**)Map of *Pf*UPF1-HA and *Pf*UPF2-HA protein-protein interactions detected by co-IP and LC- MS/MS. The dashed line represents the interaction detected in a co-immunoprecipitation experiment with *Py*ALBA4-GFP in *P. yoelii* in reference (46). Full protein names are listed in Tables 1 and 2.

A network of all protein interactions identified by co-IP is presented in Fig. 5A. Of particular interest is an apicomplexan-specific RNA-binding protein *Pf*ALBA4 (PF3D7_1347500), that co-IPed with both *Pf*UPF1-HA and *Pf*UPF2-HA. In asexual *Plasmodium yoelii*, disruption of *Py*ALBA4 led to an increase in mRNA abundance, which may imply a role for ALBA4 in mRNA decay (42). We found that *Pf*UPF2-HA also co-IPed with another putative RNA-binding protein, *Pf*GBP2 (G-strand binding protein 2; PF3D7_1006800). This protein was also identified in one out of two of the *Pf*UPF1-HA co-IP experiments (Table 1, below double line). A study in *P. yoelii* showed that *Py*ALBA4 co-IPed with *Py*GBP2, which was one of the most abundant interacting proteins (Fig. 5A, dotted line) (42). This suggests that interactions between UPF2, ALBA4, and GBP2 are conserved in *Plasmodium* (42), and in *P. falciparum Pf*GBP2 binds to RNA as well as to DNA (43). In yeast, Gbp2 is an SR (serine-arginine) protein that marks unspliced transcripts and targets them for elimination via the nuclear exosome (44). However, a recent study in *S. cerevisiae* has also shown a role for Gbp2 in mediating cytoplasmic NMD. This work showed that Gbp2 co-precipitates with all three Upf proteins and targets intron-containing transcripts for translation repression and degradation (45).

Although the core NMD interacting complex is conserved, we present evidence that suggests that canonical NMD plays little role in *P. falciparum* transcriptional regulation. The *P. falciparum* NMD core proteins impact neither steady-state transcript abundance nor asexual parasite proliferation *ex vivo*. Furthermore, degradation of intron-containing nonsense transcripts is unaffected by disruption of NMD genes, and although NMD protein orthologs interact, the canonical NMD process appears largely non-functional. Our findings corroborate other work (34) that suggests no special regulatory role for the majority of observed intron retention.

## MATERIALS AND METHODS

### Molecular biology and transfection of *Plasmodium falciparum*

Parasite lines with endogenously HA-tagged putative NMD components were generated by CRISPR using the episomal expression plasmid pHAX as a donor template. To generate pHAX, DNA sequence encoding a 3xHA tag was amplified from pGLMS-HA (47) using 3HA-FOR (GCG**ACGCGTG**CTTACCCGTACGACGTC) and 3HA-REV (GCG**TTAATTAA**TTAAGCAGCGGCATAATCTGG) primers (MluI and PacI in bold). pGLUX1-PfCentrin2-mCherry (unpublished) was digested with MluI and PacI to release the mCherry, and the PCR product was directionally cloned to generate pHAX-*Pf*Centrin2. The sequence map for pHAX-*Pf*Centrin2 is supplied in the associated GitLab repository.

For CRISPR-Cas9 editing, guide RNA (gRNA) binding sites with a protospacer adjacent motif (PAM) were selected for PF3D7_1005500 (*Pf*UPF1) and PF3D7_0925800 (*Pf*UPF2) using CHOPCHOP (https://chopchop.cbu.uib.no/) and were synthesized as crRNA without the PAM sequence (Integrated DNA Technologies) (48). Homology regions (HRs) were PCR amplified from NF54 genomic DNA with Phusion polymerase (NEB) using primers listed in Table S4. For gene disruption, two HRs 450–630 bp in length were inserted on either side of the human dihydrofolate reductase (hDHFR) drug selection cassette in pGLMS-HA (47) at the BglII and XhoI sites (HR1) and EcoRI and KasI (HR2) using the In-Fusion®HD Cloning Kit (Takara Bio). For C-terminal HA-tagging, HRs were inserted at the XhoI and MluI sites (HR1) and NcoI and KasI sites (HR2) in pHAX-*Pf*Centrin2.

HRs were no more than 30 bp from the predicted site of the Cas9-induced DNA double-strand break. Plasmids were confirmed by Sanger sequencing (AGRF Melbourne). Final plasmids (100 µg) were linearized overnight with BglII and BglI and then precipitated and resuspended in 30 µL Tris-EDTA buffer, followed by the addition of 370 µL cytomix.

Linear templates were endogenously integrated using commercial Cas9 nuclease and synthesized gRNA. To our knowledge, this is a novel technique in the transfection of *P. falciparum* and has some benefits over plasmid expression systems, as it does not require molecular cloning and plasmid preparation for Cas9 and guide components. Prior to transfection, gene-specific crRNA (100 µM in Tris-EDTA) and tracrRNA (100 µM in Tris-EDTA, catalog number 1072532) were annealed to form gRNA (Integrated DNA Technologies) by mixing 1:1, heating to 95°C for 5 min and allowing to cool to room temperature. The gRNA (3 µL) was then complexed with 2 µL *Streptococcus pyogenes* Cas9 nuclease (Integrated DNA Technologies, catalog number 1081058) at room temperature for 20 min. The resulting RNP (5 µL) was added to the linear DNA template and electroporated into 200 µL ring stage NF54-infected RBCs (~5% parasitemia) as previously described (49). After electroporation, parasites were returned to culture with new media and 300 µL uninfected RBCs. Parasites resistant to 5 nM WR99210 (Jacobus Pharmaceuticals) were observed by Giemsa smear 13–21 days following transfection. Genomic DNA was extracted from wild-type NF54, Δ*Pf*UPF1, and Δ*Pf*UPF2 parasite cultures using QuickExtract (Lucigen). The identity of transfectant parasites was verified by PCR specific for the expected parental and modified loci. PCR was performed with GoTaq (Promega) and primers listed in Table S4.

### Illumina RNA sequencing

Parasite cultures were synchronized with 5% (w/v) D-sorbitol in H_2_O in the cycle before harvest (50). Trophozoite-stage parasites (~10^10^ parasites) were isolated with 0.03% (w/v) saponin in PBS, washed with PBS, and then resuspended in 1 mL TRI Reagent (Sigma Aldrich). Chloroform (200 µL) was added to the parasite/TRI Reagent sample and was mixed by vortex for 15 s, incubated for 3 min at room temperature, and then centrifuged at 12,000*g* for 30 min at 4°C. The aqueous supernatant was removed and concentrated using a RNeasy MinElute kit (Qiagen). Samples were then treated with DNase I (Qiagen) and concentrated again using RNeasy MinElute columns (Qiagen). Library preparation and sequencing were performed by Victorian Clinical Genetics Services using TruSeq-stranded mRNA kit (Illumina) and sequenced on a NovaSeq 6,000 to a depth of 30 M reads per sample, reads were 150 base pairs, paired-end reads with three biological replicates for each sample.

### Bioinformatics analyses

RNA-seq read quality was checked using FastQC (0.11.8) before mapping of reads to the *P. falciparum* 3D7 genome (release 45) using STAR (2.7.3) (51, 52). A summary of our RNA-seq read mapping is presented in Table S5. R (version 4.0.3) was used for bioinformatics analyses (53). Differential expression of genes was tested with limma-voom using the *treat* method to determine adjusted *P*-values (requiring a log-fold change of at least 1). Genes with adjusted *P*-values < 0.05 were considered differentially expressed. Read counts used for calculating intron retention, alternative splicing analysis, including differential alternative splicing were performed using ASpli (1.10.0). Gene expression FPKM values were determined using RSeqQC (FPKM_count.py). The introduction of PTCs following intron retention was determined using a purpose-written R script. Linear mixed-effects models were fit using the R package glmmTMB (1.1.3) (54), and the R package emmeans (1.7.4–1) (55) was used to calculate means, confidence intervals and to test for differences between genotypes. All plots were generated in R using ggplot2 (3.3.3). Protein schema was generated using the R package drawProteins (1.9.1) (56). Coverage plots were created using the R package superintronic (0.99.4) (38). Analyses were performed using the Spartan High-Performance Computing system (University of Melbourne) or on personal computers. Commands and scripts used are available at gitlab.com/e.mchugh/nmd-paper. RNA sequencing data files are available on the NCBI Sequence Read Archive with the BioProject identifier PRJNA699307.

### Phylogenetic tree construction

UPF1 and UPF3b protein sequences were obtained through OrthoMCL (https://orthomcl.org/). PF3D7_0703500-related sequences were obtained by BLASTp search (https://blast.ncbi.nlm.nih.gov/) using the PF3D7_0703500 protein sequence as input and restricting results to exclude the *Plasmodium* taxon. Multiple sequence alignment was performed using the MAFFT-linsi method (v7.475) (46), and a sequence similarity plot was generated using Geneious Prime (2019.1.3) (57). Alignments were trimmed using trimAl (v1.3) (58) before construction of a maximum-likelihood phylogenetic tree with 1,000 bootstrap replicates and phylogram plotting using MEGAX (10.1.0) (59). Sequence files used are included in the associated GitLab repository.

### Statistical analysis of intron retention rate

Linear mixed-effects models were used to assess the effect of *Pf*UPF disruption, expression, and intron category on intron retention rate. One model was fitted with genotype and expression level included in the model as categorical variables, along with the interaction term. A second model was fitted with genotype and intron category included as categorical variables, also with the interaction term. In both models, biological replicate was incorporated in the model using nested random effects of day and genotype within day. Intron retention rate was log-transformed to ensure homogeneity of residual variance. All results are shown back-transformed from the log scale. Mixed models were fitted using the glmmTMB package version 1.1.4 (54) in R version 4.2.1 (R Foundation for Statistical Computing, Vienna, Austria).

### Growth analysis

Asexual parasite cultures were initiated at 1% parasitemia and 1% hematocrit in triplicate. Parasitemia was measured by flow cytometer (BD FACSCanto) every 48 h by staining with SYTO-61 (Invitrogen). Identical subculturing was performed on wild-type and gene-disrupted parasites to prevent overgrowth. Flow cytometry data were analyzed using FlowJo™ (10.6.0) software. Synchronous gametocytes were produced by the addition of spent media and 62.5 mM *N*-acetyl-D glucosamine as previously described (60, 61).

### Immunofluorescence microscopy

Infected RBCs were harvested and resuspended in PBS at 5% hematocrit. Coverslips were coated with lectin (from *Phaseolus vulgaris*; PHA-E, Sigma product L8629), washed, and infected RBCs were applied. Adhered cells were washed three times with PBS until a monolayer remained on the coverslip. The cells were then fixed for 20 min in 4% paraformaldehyde/0.008% glutaraldehyde in PBS, followed by permeabilization with 0.1% Triton X-100 in PBS for 10 min. Rat anti-HA antibody (1:300) in 3% (w/v) BSA in PBS was applied for 1.5 h, washed three times with PBS, followed by incubation with anti-rat AlexaFluor 568 (1:600) for 1 h. The nuclear stain DAPI (4′, 6-diamidino-2-phenylindole) was added prior to mounting and sealing of the slide with nail polish. Slides were imaged on a GE DeltaVision Elite Widefield Deconvolution Microscope. Deconvolved images were processed using FIJI software (62). Images stacks are presented as maximum projections and have been adjusted by cropping, adding false color and changes to brightness/contrast.

### Co-immunoprecipitation, immunoblotting, and mass spectrometry

Infected RBCs were synchronized with 5% (w/v) D-sorbitol in H_2_O, and parasites were harvested as trophozoites 72 h later. Parasites were isolated from RBCs by lysis with 0.03% (w/v) saponin in PBS on ice. Parasite pellets were then solubilized in immunoprecipitation buffer (IP buffer) containing 1% Triton X-100, 50 mM Tris-HCl, 150 mM NaCl, 2 mM EDTA, and cOmplete™ EDTA-free Protease Inhibitor Cocktail (Roche product 11836170001) for 30 min on ice. Insoluble material was separated by centrifugating twice at 13,000*g* for 10 min. The Triton X-100-soluble fraction (input) was incubated with anti-HA agarose beads (Roche product ROAHAHA) overnight at 4°C and was washed five times with IP buffer. For immunoblotting, proteins were eluted at 95°C for 10 min with Laemmli buffer containing β-mercaptoethanol. Input and eluate samples were separated on 4–15% Tris-glycine polyacrylamide gels at 200 V for 35 min. For mass spectrometry, beads were washed a further two times in 1 mM Tris-HCl (pH 7.4) before elution with 0.1% (v/v) formic acid and trifluoroethanol at 50°C for 5 min. The eluate was neutralized with triethylammonium bicarbonate, reduced with TCEP (5 mM), and then digested with trypsin for 16 h at 37°C. Samples were then analyzed by LC-MS/MS with a Q Exactive Plus mass spectrometer. Mass spectra were searched using MASCOT against a custom protein database comprising the 3D7 *P. falciparum* annotated proteome (version 43) and *Homo sapiens* reference proteome (Uniprot proteome ID: UP000005640). MASCOT searches were performed with the following parameters: MS tolerance = 10 ppm, MS/MS tolerance = 0.2 Da, cleavage enzyme = trypsin, missed cleavages allowed = 3, peptide isotope error = 0, variable modifications = oxidation (*M*), with a MASCOT decoy search performed concurrently. Proteins were considered to be enriched in *Pf*UPF1-HA or *Pf*UPF2-HA IP eluate compared to a wild-type control if there were at least two significant peptides detected in two biological replicates and (a) fivefold the number of significant peptides detected or (b) no peptides were detected in the control. False discovery rates for each experiment are presented in Table S6. The mass spectrometry proteomics data have been deposited to the ProteomeXchange Consortium via the PRIDE partner repository with the dataset identifier PXD023910.

## Data Availability

Computer scripts used for the analysis of PTCs, statistical analysis, generating figures, and Supplementary Datasets are available at GitLab. Illumina RNA-seq data have been deposited in the NCBI Sequence Read Archive BioProject under accession number PRJNA699307. Co-immunoprecipitation LC-MS/MS data are available in the PRIDE repository under accession number PXD023910.

## SUPPLEMENTAL MATERIAL

Figure S1 msphere.00233-23-s0001.pdf Confirmation of gene disruption.

Figure S2 msphere.00233-23-s0002.pdf Intron retention analysis.

Figure S3 msphere.00233-23-s0003.pdf Protein schematics.

Table S1 msphere.00233-23-s0004.xlsx Differential expression analysis.

Table S2 msphere.00233-23-s0005.xlsx ASpli analysis.

Table S3 msphere.00233-23-s0006.xlsx Co-immunoprecipitation mass spectrometry results.

Table S4 msphere.00233-23-s0007.xlsx Oligonucleotides used in this study.

Table S5 msphere.00233-23-s0008.xlsx STAR readmapping parameters.

Table S6 msphere.00233-23-s0009.xlsx Mass spectrometry search false discovery rates.

Supplemental Figure and Table Legends msphere.00233-23-s0010.docx Legends for all supplemental material.

ASM does not own the copyrights to Supplemental Material that may be linked to, or accessed through, an article. The authors have granted ASM a non-exclusive, world-wide license to publish the Supplemental Material files. Please contact the corresponding author directly for reuse.
