# Supplementary figures and images for: Nonsense-mediated decay machinery in Plasmodium falciparum is inefficient and non-essential
Source: mSphere. 2023 Jun 27;8(4):e00233-23. doi: 10.1128/msphere.00233-23 (PMC10449492; doi:10.1128/msphere.00233-23)

Figure S1

A

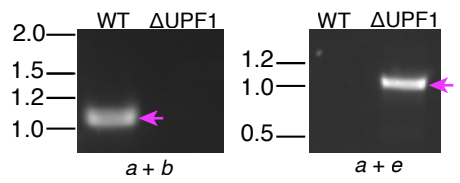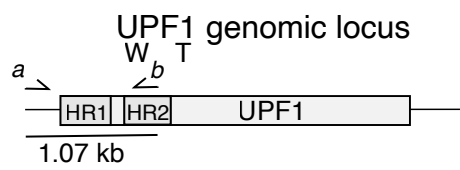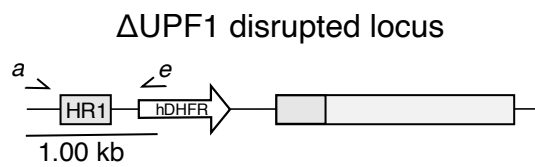

B

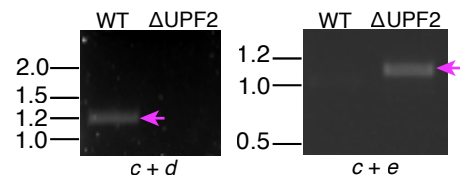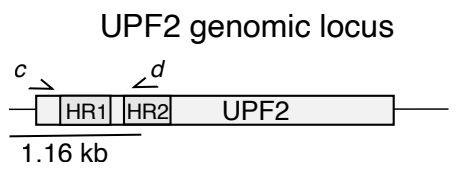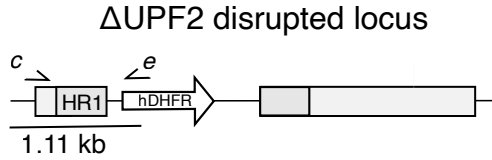

C

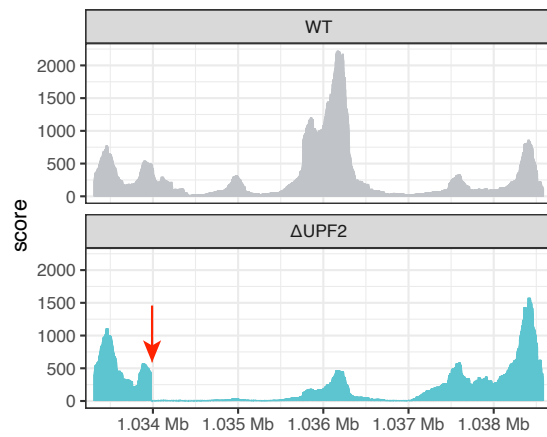

Supplement: Figure S1 — Confirmation of gene disruption. [file msphere.00233-23-s0001.pdf]

Figure S2

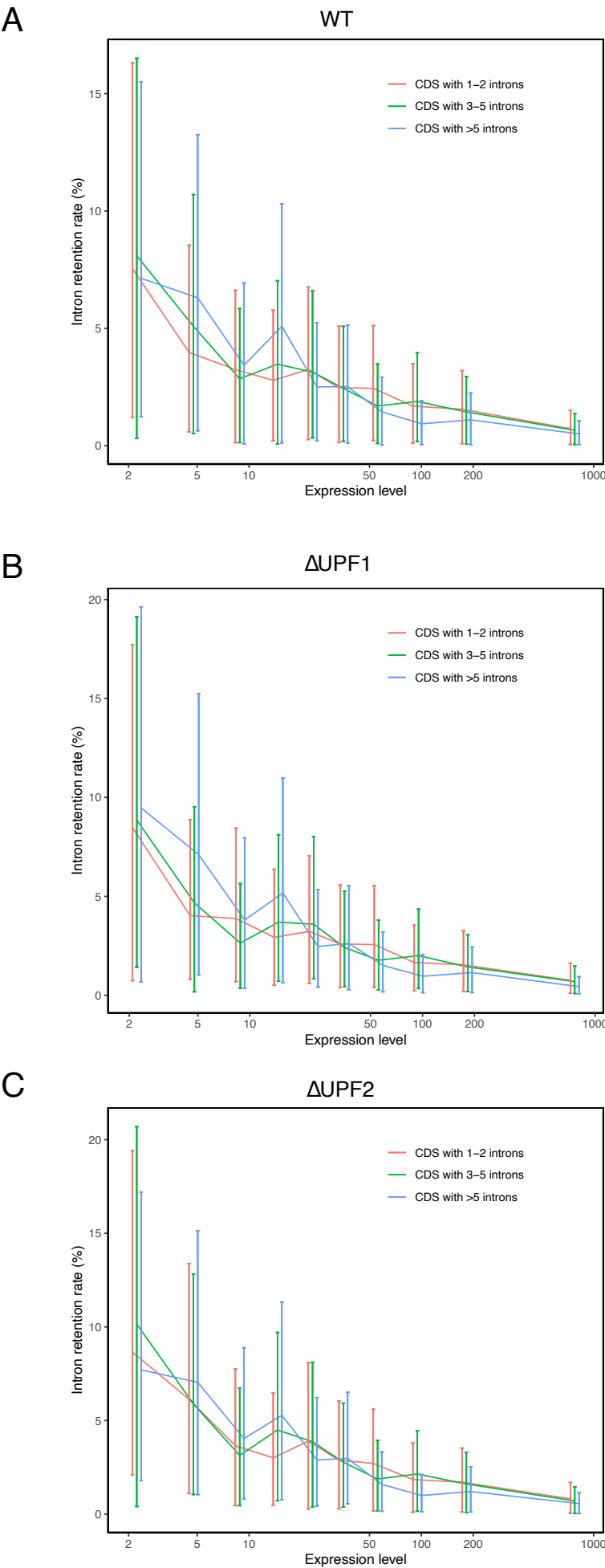

Supplement: Figure S2 — Intron retention analysis. [file msphere.00233-23-s0002.pdf]

Figure S3

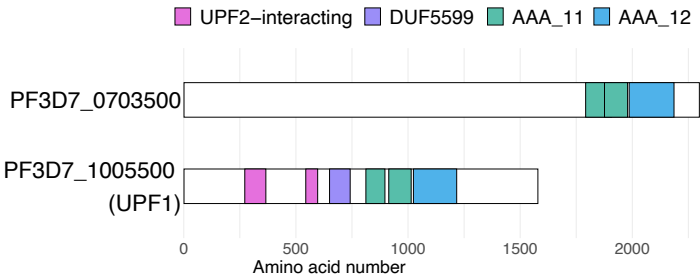

Supplement: Figure S3 — Protein schematics. [file msphere.00233-23-s0003.pdf]
