# Supplementary material for: Nonsense-mediated decay machinery in Plasmodium falciparum is inefficient and non-essential
Source: mSphere. 2023 Jun 27;8(4):e00233-23. doi: 10.1128/msphere.00233-23 (PMC10449492; doi:10.1128/msphere.00233-23)
Supplement: Supplemental Figure and Table Legends — Legends for all supplemental material. [file msphere.00233-23-s0010.docx]

**Supplementary Figures**

Figure S1
PCR confirmation of gene disruption in Δ*Pf*UPF1 and Δ*Pf*UPF2 parasites.
(A) A PCR product (1.07 kb, primers a + b) is amplified from the *Pf*UPF1 locus with WT parasite DNA but not Δ*Pf*UPF1 parasite DNA. An amplicon (1.00 kb, primers a + e) from the disrupted Δ*Pf*UPF1 locus, resulting from integration of the hDHFR cassette, is amplified only from Δ*Pf*UPF1 parasite DNA. PCR products are indicated with pink arrows. (B) An amplicon (1.16 kb, primers c + d) resulting from the *Pf*UPF2 genomic locus is only amplified from WT parasite DNA. Note that primer ‘c’ anneals upstream of the 5’ homology region. A product (1.11 kb, primers c + e) amplified from only Δ*Pf*UPF2 parasite DNA confirms disruption of the CDS with the hDHFR cassette. Primer sequences are listed in Table S9.

(C) Coverage plot showing RNA-seq reads mapped to the *Pf*UPF2 genomic locus from WT and Δ*Pf*UPF2. The gRNA-Cas9 target site where the drug cassette insertion occurred in Δ*Pf*UPF2 parasites, is indicated with a red arrow.

Figure S2
Introns (n = 7523) were grouped into 10 equal-sized bins based on expression level (WT FPKM) and for (A) WT and (B) Δ *Pf*UPF1 and (C) Δ*Pf*UPF2 the IR rate was computed globally within each bin as a proportion. CDS 1-2 = 1969 introns, CDS with 3-5 = 2222 introns, CDS with >5 introns = 3332. Error bars represent 95% confidence interval.

Figure S3
Protein schema showing Pfam domains in PF3D7_0703500 and *Pf*UPF1. AAA_11 and AAA_12 are AAA ATPase domains associated with ATP-dependent helicase activity. DUF6699 (DUF = domain of unknown function) is a domain also present in the *Homo sapiens* UPF1. The N-terminal UPF2- interacting domain contains zinc binding motifs which interact with UPF2.

**Supplementary Tables**Table S1: Results from differential gene expression analysis performed using limma.
Table S2: Intron read counts and analysis performed using ASpli.
Table S3: Co-immunoprecipitation mass spectrometry peptide search results.
Table S4: Oligonucleotides used in this study.
Table S5: Read mapping parameters used for the STAR algorithm.
Table S6: False discovery rates for mass spectrometry analysis.
